# Extrauterine Growth Restriction and Optimal Growth of Very Preterm Neonates: State of the Art

**DOI:** 10.3390/nu15143231

**Published:** 2023-07-21

**Authors:** Antonios K. Gounaris, Rozeta Sokou, Eleni A. Gounari, Polytimi Panagiotounakou, Ioanna N. Grivea

**Affiliations:** 1 Neonatal Clinic-NICU, University Hospital of Larissa, 413 34 Larissa, Greece; ioanna.grivea@gmail.com; 2 Neonatal Clinic-NICU, Nikea General Hospital “Agios Panteleimon”, 184 54 Piraeus, Greece; sokourozeta@yahoo.gr (R.S.); ppppolytimi04@gmail.com (P.P.); 3 East Sussex Hospitals NHS Trust, Hastings TN37 7PT, UK; elenigounari@gmail.com

**Keywords:** very premature neonates, extrauterine growth restriction, postnatal growth failure, optimal growth, nutrition, feeding policies

## Abstract

Over the last few decades, there has been an ongoing debate over both the optimal feeding mode for very premature neonates (VPN) as well as what their optimal growth should be. Despite the American Academy of Pediatric declaring since 1997 that the growth of VPN should follow the trajectory of intrauterine fetal growth, differences of opinion persist, feeding policies keep changing, and the growth and development of VPN remains extremely variable not only between countries, but even between neighboring neonatal units. Even the appropriate terminology to express poor postnatal growth (extrauterine growth restriction (EGR) and postnatal growth failure (PGF)) remains a subject of ongoing discussion. A number of recent publications have shown that by implementing breast milk fortification and closely following growth and adjusting nutrition accordingly, as per the consensus guidelines of the major Neonatal Societies, we could achieve growth that closely follows birth centiles. A recent position paper from EPSGAN recommending targeted nutritional support to cover the energy and protein deficits sustained by VPN during periods of critical illness further strengthens the above findings. Conclusion: We can promote better growth of VPN by ensuring a stable administration of sufficient calories and protein, especially in the first 2 weeks of life, implementing breast milk fortification, covering energy and protein deficits due to critical illness, and increasing feeding volumes as per the latest guidelines. The adoption of universal protocol for nutrition and growth of VPN is essential and will enable better monitoring of long-term outcomes for this population.

## 1. Introduction

The third pregnancy trimester is a period of rapid fetal growth and development. The normal development of the fetus is disrupted during premature birth, and both intrauterine and extrauterine growth affect the long-term health of very premature neonates (VPN). In the last three decades, there has been ongoing discussion on what constitutes optimal growth for VPN. There are different opinions expressed and even different definitions/interpretations of the terms intrauterine growth restriction (IUGR), extrauterine growth restriction (EGR), and postnatal growth failure (PGF), resulting in the absence of a universally approved pathway for the nutrition and growth of this population.

Two recent major publications have put the issue of those definitions in the forefront, declaring the terms EGR and PGF as misnomers that need to be replaced [1] and suggesting new centile charts for VPN [2]. The esteemed authors of both articles cite multiple important reasons for their approach and particularly the risks that may arise from providing “excessive” nutrition in an effort for VPN to grow close to their birth centile at all costs.

Despite that, other recent publications [3,4] shed a different light on the issue as they achieved a significant reduction in VPN less than 10th centile at 36 weeks corrected age, without administering calories or protein in excess of what is recommended in the neonatal guidelines. It is evident from the above that it is important for the discussion on feeding policy and VPN growth to include the utility or not of the term EGR. It is noteworthy that although both the terms EGR and PGF are used to describe growth restriction, EGR, which implies a cut-off value at a determined point in life, refers to growth restriction until 40 weeks and is preferable to PGF, which implies a difference in growth velocity during an unspecified period that may extend to 1 year of life. Looking through the literature, it appears that the term EGR, defining neonates that are below the 10th percentile of intrauterine growth expectation, is too broad and can include neonates for whom the difference between birth and 36 weeks corrected gestational age (CGA) can range from −0.5 SD or −1 SD up to −2 SD. Clearly, neonates in the extremes of the EGR spectrum have vastly different risks of adverse outcomes. In this review, we will attempt to examine the validity, usefulness, and adequacy of the term EGR, and what would be considered optimal growth for VPN.

## 2. Feeding Policies Pre-2000 and Effect on VPN Growth and Prognosis

We need to remember that the term EGR was not commonly used prior to the year 2000. During that period, the initial enthusiasm from the widespread use of antenatal steroids and postnatal surfactant started to wane on the realization that the huge increase in survival, even at the extremes of viability, was not followed by an equivalent reduction in VPN with neurodisability. On the contrary, it resulted in an increase of the percentage of VPN surviving with significant neurodevelopmental abnormalities [5].

There were two further notable developments at that time. Firstly, it was established that the feeding policies, especially in terms of the amount of protein given, resulted in widespread growth restriction of VPN as demonstrated in the large NICHD/NNR study [6] where 97% of VP neonates were below 10th centile at 36 weeks CGA. Secondly, in the late 1990s, large prospective and retrospective studies showed that IUGR increased the risk of metabolic syndrome in later life [7]. Following those developments, the term EGR started coming into widespread use.

**Figure 1:**
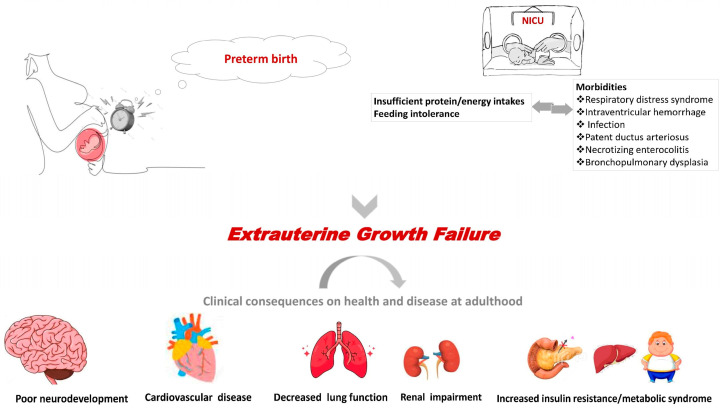
Clinical consequences of extrauterine growth failure on health and disease at adulthood.

Many articles looking at the effects of different feeding strategies in the first and second era of neonatology found that prematurity and growth restriction before 40 weeks CGA increased the risk of adverse neurodevelopmental and metabolic outcomes (Figure 1) [8,9,10,11,12,13]. The understanding that the main reason for EGR in VPN was that nutrients and especially protein was less compared to the nutrients received through the placenta at different gestational ages led to a reconfiguration of feeding policies.

## 3. The Effect of Early “Aggressive” Feeding Strategies Post-2000

In 2002, Ziegler et al. first used the term “aggressive” feeding to describe a feeding protocol that was in his opinion closer to the nutrition received by the mother through the placenta [14]. In the following decade, more authors and pediatric societies adopted a similar approach, suggesting feeding policies with more nutrients and focusing on protein content. The outcome of these policies was the marked reduction in the percentage of VPN with EGR, although it still remained high at around 50% [15]. In a more recent study, the percentage of VPN with EGR at 36 weeks CGA in 11 European countries showed a wide variation from 24% in Sweden to 60% in Portugal [16].

A large study of >90,000 VPN born between 2007 and 2018 showed that the mean value for weight and head circumference (HC) at 36 weeks CGA was >−1 SD for all gestational ages <32 weeks, and for neonates less than 28 weeks GA it reached −2 SD. It is clear that there are big variations in practice between different countries and between NICUs within the same country [2]. Embleton [17] asked in 2001 whether EGR is unavoidable, and this question still remains relevant today: is EGR unavoidable and, if so, to what degree?

A meta-analysis [18] and studies looking at associations between EGR and long-term prognosis of VPN born after 2000 have found a negative effect [19,20,21,22]. A recent study (LEMON study) compared monochorionic diamniotic twins from uncomplicated pregnancies that had differences in their intrauterine growth. Infants that had significant growth restriction compared to their twins had statistically significant risk of having lower IQ and exhibit moderate neurodevelopmental disorders [23].

In a very recent study, higher neonatal growth velocity in children born less than 29w GA was associated with modestly higher cognition and language score at 18–22 months CA [24]. These results were more profound among those born with the lower weight for gestational age, emphasizing the importance of postnatal growth in this population. It is clear that growth restriction of VPN either intrauterine or extrauterine can adversely affect their outcome, and the question posed by Embleton requires an urgent answer.

## 4. Factors Influencing Nutritional Supply and Growth of Preterm Infants

It is evident that prior to 2000, EGR of VPN was due to feeding policies with inadequate calorie and protein intake. The question remains, though, why the subsequent increases in nutrients provision did not solve the problem. In an effort to define the issue, some recent studies and meta-analyses have looked in more detail into the energy and nutrients provision of VPN. They found that infants with EGR were receiving fewer calories and less protein than the recommended from the Neonatal Societies’ guidelines for variable periods of time and for a variety of reasons, mainly during the transition phase from the parenteral to enteral feeding [25,26,27]. Another possible cause is the lack of breast milk fortification.

### 4.1. Breast Milk Fortification

Is breast milk fortification necessary throughout admission? This remains controversial. A large study of over 45,000 VPN showed that of the VPN receiving breast milk, only 45.3% received fortification of some degree [28].

The results of some recent studies may aid decision making. Breast milk of mothers of premature neonates contains extra protein compared to that of term neonates only for the first month. The peak protein content is at 2 weeks (approx. 1.9 g/100 mL mean value) with subsequent gradual decrease in the following 2 weeks and without birth gestational age further affecting it [29,30]. In 2019, Li et al. [31], comparing VPN that received preterm breast milk with ones that received preterm breast milk with preterm formula, showed that the latter had better growth at 40 weeks CGA (mean difference 283 g; (95% CI: 121.6–445.6) without difference in body fat content on whole body MRI at 37–44 weeks. These results mean that the difference was down to the better growth of other vital tissues (lean tissue). The fortification rate of preterm breast milk was almost similar in all groups. In a recent paper, Perrin et al. [32] found that individualized fortification of human milk prevented postnatal weight loss in most infants and supported HC growth.

A 2023 study from Embleton et al. [33] showed that cow’s milk-based fortifier did not adversely affect the gut microbiome when compared to human milk-based fortifier. Reservations on the use of breast milk fortifier focusing on intolerance to cow’s milk and the risk of NEC do not appear to be substantiated on a meta-analysis of the data, which actually showed moderately better growth on VPN receiving fortifier [34], and a further recent study considers breast milk fortification necessary for adequate growth of VPN [35].

In our opinion and in view of those results, fortification of breast milk is necessary, as unfortified breast milk is not sufficient in the majority of cases, even in the first 4 weeks, to cover the nutritional needs of VPN.

### 4.2. VPN with Major Morbidities and Growth

Major morbidities (BPD, IVH, NEC, ROP, etc.) are complications that increase the risk of EGR. In the large study by Greenbury et al. [2], VPN with major morbidities had significantly restricted growth compared to the ones without. A likely contributing factor is the difficulty in administration of nutrients in this population. In a study by Milanesi et al. [36], VPN that eventually developed BPD did not receive the correct ratio of calories to protein for periods spanning from birth up to 4 weeks of age.

Ehrenkranz et al. [37] showed a correlation between major morbidities such as BPD and the development of VPN, considering EGR as a major factor underlying both. In another study, VPN with BPD that received an intense and targeted feeding regime and had similar growth to VPN without BPD did not show significant difference in their respiratory function at 8 years compared to VPN without BPD and term controls [38]. Groene et al. [39] showed that monochorionic twins with selective fetal growth restriction had significant increase in the prevalence of BPD despite the lowest incidence of respiratory distress syndrome compared to the larger co-twin, in spite of their identical genetic makeup and maternal risk factors, essentially in a form of a “natural experiment” that showcases the link between growth restriction and lung function. The above observations pose a question. Is a different feeding protocol needed for VPN with major morbidity in order to prevent further growth restriction and, if so, from what point in their disease?

Our opinion is that VPN with major morbidities should receive intense nutrition, to minimize their postnatal growth difference to those without major morbidities [40].

## 5. Feeding Policies and Reducing the Percentage of VPN with EGR

Several neonatologists from different countries have implemented feeding policies that have succeeded in reducing the percentage of VPN with EGR [3,4,41,42,43]. Some have achieved that by monitoring growth and correlating it with number and duration of interruptions in enteral feeding [41], others by providing higher protein content in the first 2 weeks of life [3,4], or by providing larger quantities of milk [42], with some giving more than 200 mL/kg/day of milk [43,44,45]. A common thread in most of those publications is breast milk fortification, close monitoring of growth at least on a weekly basis, and adjusting nutrition, according to the guidelines of Neonatal Societies, in order to achieve growth close to birth centiles.

In a 2022 publication from Rossholt et al. [46], close monitoring of growth resulted in only 3% of VPN having >−1 SD deviation from their birth centile at 36 weeks CGA. None of the above studies have recorded any instances of neonates receiving calories or protein in excess of the recommended guidelines.

## 6. And Now What: Current and Future Demands

Despite many different feeding protocols that promote growth and reduce EGR, it remains difficult to achieve that goal more broadly due to the absence of universally accepted policies and the variability between different countries and different NICUs. A characteristic example is a cluster of 10 different enteral feeding policies in one country reported by Greenbury et al. [28].

There are two distinct themes arising from the data above. Firstly, there is clear evidence that growth restriction of VPN up to 40 weeks CGA, despite the changes in feeding policies post 2000, increases the risk of metabolic syndrome and neurodevelopmental disorders. Whilst correlation does not equal causation, this evidence should not be ignored. Secondly there are NICUs that, by closely following the recommendations from the neonatal societies, have achieved reducing the percentage of VPN with EGR to less than 10%.

Two recent position papers form ESPGHAN can help in providing some answers to the questions we have posed above (Table 1) [47,48]. According to the 2021 EPSGHAN position, premature neonates with critical illness should have the energy and protein deficit sustained during that period replaced during the recovery phase in order to achieve catch-up growth. This, according to the authors, can be achieved by increasing calories during the recovery phase up to 160 kcal/kg/day, protein up to 4.5 g/kg/day, glucose up to 12.5 g/kg/day, and fat up to 8 g/kg/day for as long as required in order to replace nutrient/energy deficits sustained during the acute illness phase [47].

The ESPGHAN position paper published in 2022 [48] alters previous guidelines published in 2010 [49] and for the first time recommends increasing calories up to 160 kcal/kg/day, protein up to 4.5 g/kg/day and milk up to 200 mL/kg/day or higher in some case of enterally fed VPN, in order to achieve improved growth. Those two ESPGHAN positions acknowledge that the period of up to 40 weeks CGA is critical for neonatal development and promote catch up growth following periods of growth restriction due to VPN complications.

Coming back to the initial question regarding EGR, the issue is not whether the term itself is correct. In our opinion, the issue is that this one term is too broad and can encompass a very wide range of neonates (from Z score > −0.70 up to >−1.70) that are likely to have vastly different risk profiles and sustain very different outcomes. We feel that a more nuanced approach that takes into account the vastly different risks at different points of the EGR spectrum is required.

What could be the target today? What level of VPN growth is both safe and realistic to achieve?

Based on the recent ESPGHAN recommendations and other recent publications, and using the traditional definition of EGR (<10th centile), it is achievable today for neonatologists to aim to limit the percentage of VPN with weight < 10th centile at 36 weeks or at discharge to around 10% of total [3,4]. In a 2006 study, it was shown that growth on the 10th centile for weight and HC at 36 weeks CGA did not increase the risk of neurodevelopmental disorders [50]. On that basis, a growth for VPN to achieve weight and HC > 10th centile at 36 weeks CGA is considered safe. Expressing EGR as a z score, this target would be a difference from birth weight centile of up to −0.70 or up −0.80 at 36 weeks CA [3,51]. For neonates born at less than 26 weeks GA, this difference could be a little bit higher but less than −1 z score.

Regarding the question of a universal feeding policy, in our view, the close monitoring of VPN growth and strict adherence to the latest ESPGHAN guidelines, especially the ones concerning catch-up growth following acute illness (Table 1), will significantly contribute to reducing neonates with growth restriction.

After all, in the era of constantly rising rates of extremely premature survivors, a universal definition EGR and relevant robust guidelines on neonatal feeding are both highly desirable.

In conclusion, we feel that the term EGR is useful, but it needs to be better defined in order to express its real effect in the early and late neonatal prognosis. From the reported data, it is obvious that very premature neonates require close monitoring of their growth and adequate and reliable administration of nutrients based on the latest guidelines, both via the parenteral and enteral route, including breast milk fortification and especially in the first 2 weeks of life. Any energy or nutrient deficits sustained during periods of acute illness or significant co-morbidities should be replaced within the 40 weeks GCA. Studies with nutritional practices that succeeded growth of VPN close to the birth centiles could today be the guide for the implementation of universal common feeding protocols and growth for the VPN.

## Figures and Tables

**Table 1:** ESPGHAN recommendations regarding energy and macronutrient requirements in preterm neonates.

Energy/Macronutrient	ESPGHAN 2022 Recommendations for Enteral Nutrient Intake; Embleton [48]	Energy/Nutrient Requirements in Critically Ill Neonates; Moltu [47]			
			Early Acute	Late Acute	Recovery
**Energy (kcal/kg /day)**	115–140 (160)	Enteral	40–55	70–95	110–160
		Parenteral	40–55	60–80	90–120
**Fluid, mL/kg/d**	135–200 (>200)	_	_	_	_
**Glucose (g/kg/d)**	11–15	Enteral	5–8	7–11	11–15 (18)
		Parenteral	5–8 (10)	7–10 (12)	11–14 (17)
Protein (g/kg/ day) *	3.5–4.0 (4.5)	Enteral	1.0–2.0	2.0–3.0	3.5–4.5
		Parenteral	1.0–2.0	2.0–3.0	2.5–3.5
**Lipids (g/kg/ day)**	4.8–8.1	Enteral	2.0–3.0	3.0–6.0	5.0–8.0
		Parenteral	1.0–2.0	2.0–3.0	3.0–4.0

Data in brackets represent upper intake; * to facilitate protein utilization, a non-protein energy to protein ratio of >25 kcal/g protein or a protein to energy ratio of 2.8–3.6 g/100 kcal is recommended.

## Data Availability

Data are contained within the article.
